# Twin-Screw Extrusion as Hydrothermal Technology for the Development of Gluten-Free Teff Flours: Effect on Antioxidant, Glycaemic Index and Techno-Functional Properties

**DOI:** 10.3390/foods11223610

**Published:** 2022-11-12

**Authors:** Ana Belén Martín-Diana, Belén Blanco Espeso, Ivan Jesus Jimenez Pulido, Pedro J. Acebes Martínez, Daniel Rico

**Affiliations:** 1 Agrarian Technological Institute of Castilla and Leon (ITACyL), Ctra. Burgos Km 119, Finca Zamadueñas, 47071 Valladolid, Spain; 2 Technological Centre Cartif. (Cartif), Parque Tecnológico de Boecillo, parcela 205, 47151 Boecillo (Valladolid), Spain

**Keywords:** teff, rice, extrusion, twin screw, antioxidant, glycaemic index

## Abstract

Gluten-free products (GFP) currently are the fastest-growing category of baked goods probably due to the high worldwide incidence of celiac disease (CD). Refined rice is one of the most used cereal flour for GFP development, due to its high content in starch and good technological aptitude. However, its low content in fibre, protein and minerals has awakened a recent interest as alternative to balance the GF flour formulas. Teff is a cereal with high levels in fibre and antioxidants compounds but the lack of gluten results in very limited techno-functional properties. Extrusion is a thermal technology that allows to combine flours, overcoming negative impacts on quality characteristics. This study evaluated the effect of twin-screw extruder on rice-teff (white and brown) mixtures with different teff concentrations (25, 50 and 75%) on their antioxidant, glycaemic index and techno-functional properties. The results showed than the high shear–temperature process produced important modifications on the flour, which were confirmed using scanning electron microscopy (SEM). Significant increases in total dietary fibre (16 to 100% increase) were observed in teff containing flours, due to carbohydrate–lipid–protein complexes, which lead to resistant starch, with no significant increase in rice flour. Hydration and pasting properties were significantly (*p* > 0.05) affected by extrusion, and the effect was related to the concentration of teff used. The thermal process showed a decrease in total phenol (TP) content for rice; however, extrusion enhanced the release of total phenol in rice-teff blends, which was reflected on the antioxidant activities of blend flours, especially those prepared with brown teff.

## 1. Introduction

Celiac disease (CD) is a chronic autoimmune gastrointestinal pathology that appears in genetically predisposed population with environmental and immunological components. CD causes atrophy of the intestine microvilli, resulting in malabsorption of nutrients among other problems. Celiac disease affects 1:100–1:200 worldwide, although many remain undiagnosed. It is caused by intolerance to gluten, a protein that is present in wheat, barley, rye and some varieties of oats, as well as their hybrids [1].

Gluten is composed of two fractions of proteins: prolamins and glutenins. Most toxicity studies have been carried out with prolamins, although glutenins may also have immunostimulatory effect on celiac patients [2]. Prolamins receive different names depending on where they came from, in wheat are known as gliadins, in barley hordenins, secalins in rye and avenins in oat gluten. The prolamins gliadin, hordein and secalin have similar structural conformation, however, avenins are structurally quite different, which is the main reason why many tests cannot identify and quantify them [3]. Probably, this structural difference also may explain why oats are better tolerated in most people with CD, moreover it is important to consider that prolamins in oat only make up about 10–15 per cent of total protein content, compared to 40–50 per cent of the total protein content in wheat [4,5].

A gluten free diet (GFD) is the standard care for patients with CD, including silent forms such as allergy, intolerance or sensitivity to gluten due to non-identified causes [6]. Gluten-free products (GFPs) are initially designed for people with these conditions; however, there is an increasing number of consumers interested in these products, probably due to the perception of gluten free products as healthier, less caloric and more natural than regular products, even though their self-reported knowledge about celiac disease and/or GFPs is scarce [7].

One of the main problems of GFD is the low adherence of patients to it, due in part to GFPs poor organoleptic quality. Nevertheless, poor nutritional profiles of GFPs may also cause nutritional deficiencies in consumers following a GFD, which can increase the risk of poor bone health, pregnancy outcome and potential lymphoma development [6,8,9]. However, it is important to highlight that the adherence to this type of diet in population without any CD diagnosis or symptomatology is also questioned by the medical community, since there are different studies that show evidence of potential harms of a GFD including nutritional deficiencies, economic costs and psychosocial aspects [10]. In addition, gluten is difficult to replace as an ingredient, due to its techno-functional properties, and frequently gluten-free doughs result difficult to handle due to lack of cohesiveness, elasticity, expansion and baking quality [11].

Rice is probably the most popular cereal used in gluten free factory due to its excellent techno-functional and hypoallergenic properties, absence of colour, bland taste and low levels of prolamins [12]. However, their rice has a low protein, fibre and mineral content, making it necessary to combine it with other raw materials for gluten-free product development [13].

Teff [*Eragrostis tef (Zucc.)* Trotter] is an annual crop from Poaceae (grass) family. Regarding its chemical composition, starch comprises up to 70% of dry matter, followed by protein (10.5–12.8%), fibre (9.8%) and with a relatively high content of lipids (3.7%) when compared with cereals, where 84% correspond to unsaturated fatty acids [14,15,16,17]. The high nutritional value of teff is due, in part, to a relatively large germ compared to other cereal seeds [18]; due to its small size the flour is only produced using the whole grain [14]. Different authors have investigated the nutritional [19] and rheological [20] properties of teff. In addition, antioxidant properties of teff have been reported [17,20]. Probably all these benefits would explain this cereal’s increasing popularity [21].

Teff is rich in several polyphenols, including catechin, ferulic and rosmarinic acids as the more abundant in the soluble fraction, and ferulic, rosmarinic and p-coumaric acids are mainly found as bound phenolics [21]. Furthermore, teff has relatively higher crude fibre content compared to other common grains and lower GI. Therefore, teff-based foods are expected to have outstanding contribution to the prevention and amelioration of diabetes [22].

Teff is consumed as a whole grain and has a similar starch and protein structure to sorghum, which is characteristic by the presence of compact aggregates in protein bodies around the starch granules. One of the main drawbacks of teff and sorghum proteins is the lack of unique functional properties of wheat proteins in terms of viscoelastic dough formation. On the other hand, the significant phytic acid content of teff limits its nutritional value related to mineral content. For this reason, teff flour benefits from pre-treatment application, which facilitates its transformation into food; for example, in Ethiopia it is usually fermented to produce injera [21]. Different studies have mentioned that hydrothermal technologies are an interesting approach to facilitate its consumption [23].

There are few investigations that correlate the hydrothermal treatments, as extrusion for example, with the quality of gluten-free products, specially focused on teff [17,23]. Stojceska et al. [24] studied gluten-free products made from vegetables, fruits and teff, and concluded that extrusion technology has the potential to increase the levels of total dietary fibre in the final product. Jafari et al. [25], reported that dough containing extruded sorghum enhanced bread making. Other crops, such as wheat, oat, corn or rice, have been more extensively studied regarding the effects of extrusion on functional properties at a molecular level [23].

Extrusion technology has an important impact on food industry due its highly versatile process allowing a wide variety of products due to its effect on proteins and starch which, at the end, modifies functionality, texture, and other properties [26]. Several studies point out that extrusion technology in flours produce modifications at a molecular level, revealing interesting changes in its properties after processing [27,28,29,30,31,32]. Different processing conditions produce different modifications on the product depending on parameters as temperature and shear, mainly, and others as pressure, speed and water content [23]. Extrusion has the ability of increasing the digestibility of starch and protein [33] and breaks down mineral and antinutrients complexes by hydrolysis, increasing the mineral bioavailability and vitamins, and reduces antinutrient which could impact negatively on the nutritional profile of the final product after extrusion [34].

One of the most affected macromolecules by extrusion is the starch; high temperature application in hydrothermal treatments, exceeding the point in which flour starch gelatinisation is produced, leads to an irreversible rupture of starch. Therefore, starch reduces its crystalline structure, and amylose molecules are released and amylopectin chains are consequently degraded [31]. This phenomenon is known as dextrinisation [23,31]. One of the consequences of extrusion is the increase of flour solubility due to the starch swelling. Pasting properties can also be affected by extrusion through starch gelatinisation [23,31]. Extrusion affects other macromolecules such as proteins, resulting in denaturation, enzyme (in)activation, and Maillard reactions [23,31]. Protein modifications produce association, dissociation and aggregation of subunits by non-covalent and covalent bonds, which affect different aspects of the final raw material [29].

Starch modifications after extrusion increase the presence of oligosaccharides and the number of hydroxyl groups available to form hydrogen bonds with proteins, affecting emulsifying capacity and stability of flours [28,35]. The chemical susceptibility of starch granules towards hydrolytic enzymes could be improved with gelatinisation. All these modifications affect protein digestibility and total dietary fibre solubilisation [27]. Nikmaram et al. [30] also described the influence of extrusion on the inactivation of lipidic oxidation enzymes and the reduction on antinutritional factors and microbial load. Therefore, hydrothermal treatments such as extrusion could be a beneficial strategy to obtain modified starch with different functionality, as it is currently achieved through chemical reactions [23].

Although different authors have studied the properties of teff flours and the use of hydrothermal treatments in those type of materials, few studies have reported the effect of extrusion on nutritional, techno-functional (hydration and pasting properties) and antioxidant properties. This study aims to evaluate the effect of twin-screw extruder on rice-teff (white and brown) mixtures at different teff concentrations (25, 50 and 75%) on flours as an ingredient, and the impact on their healthy (antioxidant and glycaemic index) and techno-functional properties to demonstrate the need to implement the use of technology such as extrusion to facilitate the development of new alternatives to conventional nutritional and functional balanced gluten free products.

## 2. Materials and Methods

### 2.1. Chemicals

The following compounds were used in the analysis: Folin–Ciocalteu (FC) reagent, gallic acid (GA), and 6-hydroxy-2,5,7,8-tetramethyl-2-carboxylic acid (Trolox), 2,2′-azinobis 3-ethylbenzothiazoline-6-sulfonic acid (ABTS^•+^), 2,2′-diazobis-(2-aminodinopropane)-dihydrochloride (AAPH), fluorescein, 2,2-diphenyl-1-picrylhydrazyl (DPPH^•^), were obtained from Sigma-Aldrich, Co. (St. Louis, MO, USA).

### 2.2. Material

White and brown teff (Eragrostis tef, Zucc.) flours were kindly provided by a local company Salutef (Palencia, Spain) and rice (Oryza sativa) flours by a local milling company Molendum Ingredients (Zamora, Spain).

### 2.3. Experimental Design

White and brown teff (WTF and BTF) flours were mixed with rice flour (RF) and considered as native flours (M1 to M7). Table 1 shows the percentages of rice and teff of different blend mixtures. Concentrations higher than 50% of teff flours were not considered due to excessive bitterness reported by the authors in previous studies [17].

All the native blends (M1–M7) were extruded and milled and the corresponding extruded samples E1–E7 were obtained.

### 2.4. Extrusion Process

**Figure 1:**
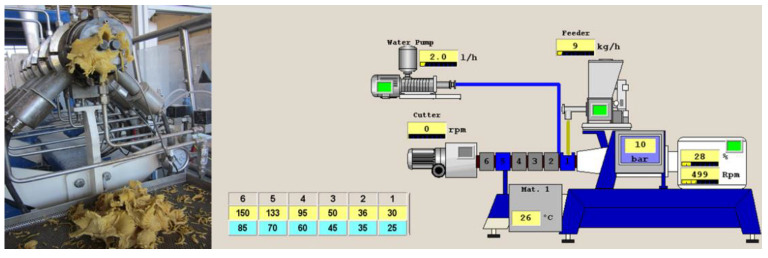
Twin–screw extruder (**left**) and display of process parameter monitoring (**right**).

Blend flours (Table 1) with no water addition were extruded using a twin-screw pilot-scale extruder (Figure 1). Extrusion was carried out using a Evolum25 twin–screw extruder (Clextral, Riez 42,702 Firminy Cedez, France) with co-rotating and closely intermeshing screws and a run capacity of 25 kg of feed/h was used. The extruder was equipped with six-barrel sections, each of 100 mm in length with independent heating, and the total configured screw length (L) was 600 mm. The screw diameter (D) was 25 mm, and the L/D ratio was 24:1.

The material was fed into the extruder using a LWFD5-20 volumetric feeder (K-Tron Corp., Pitman, NJ, USA) at a medium rate of 9 kg h^−1^. The temperature profile was set up at 30, 35, 46, 88, 126 and 141 °C for barrels 1 to 6, respectively. Extruder screw speed was kept constant at 500 rpm and water was added in a rate of 2 kg h^−1^. Extrusion conditions were selected from previous trials in order to obtain an optimised flow of the flour (Figure 1).

### 2.5. Proximal Analysis

The following compositional parameters were analysed: total fat content was measured using dried samples extracted with petroleum ether (BP 40–60 °C) for 4 h using a Soxtec fat extracting unit (AOAC 2005, method 2003.05) [36]. Moisture content was evaluated gravimetrically by drying samples at 100 °C for 24 h. Total protein content was measured by the Dumas method, AOAC method 990.03 [36] in an elemental analyser and a conversion factor of 5.7 was used to calculate the protein content from total nitrogen values. Ash values were determined by sample incineration in a muffle furnace at 550 °C for 5 h (AOAC 2005, method 923.03) [36]. And carbohydrate content was estimated as the difference between 100 and the sum of protein, fat and ash content. Total dietary fibre (TDF) content was evaluated using TDF100A-1KT assay kit (Sigma, St. Louis, MO, USA), in accordance to the manufacturer’s instructions, based on AOAC method 985.29 [36]. All results were corrected for moisture content and expressed as g 100 g^−1^ of dry matter (d.m.). Parameters were evaluated in duplicate.

### 2.6. Colorimetric Study

Lightness (L*), redness (a*) and yellowness (b*) were measured using a colorimeter (Colour Quest XE Hunter Lab, Northants, UK). The illuminant was D65 (colour temperature of 6504 K), and the standard observer was 10°. The colorimeter was standardised using a light trap and a white calibration plate. Measurements were carried out in triplicate and taken directly on the samples.

### 2.7. Techno-Functional Properties

#### 2.7.1. Hydration Properties

Hydration properties were analysed in the extruded and non-extruded flours in order to observe the effect of extrusion technology. Swelling volume (SV) and water holding capacity (WHC) were determined following the method by Nelson [37]. Briefly, 2 g of sample were mixed with 20 mL of distilled water and kept at room temperature. After 24 h, the supernatant was removed, and the weight and volume of the remaining hydrated sample were recorded. WHC was defined as grams of water retained per gram of solid and SV as total volume of the swollen sample per gram of dry weigh [37].

Water absorption capacity (WAC) and oil absorption capacity (OAC) were analysed following the method of Beuchat [38]. Two grams of sample were mixed with 20 mL of distilled water, for WAC, or oil (refined sunflower oil) for OAC determination. Samples were kept at room temperature for 30 min, and mixed occasionally with vortexing. Afterwards, samples were centrifuged for 30 min at 3000× *g* and supernatant was removed. WAC and OAC parameters were expressed as grams of water or oil retained per gram of flour.

Water absorption index (WAI), water solubility index (WSI) and swelling power (SP) were calculated following the method described by Kaushal et al. [39]. Samples (2.5 g) of flours (w_0_) were mixed with 30 mL of distilled water and heated in a water bath at 90 °C for 10 min. After cooling at room temperature, samples were centrifuged at 3000× *g* for 10 min; supernatant was dried in an oven at 110 °C overnight (w_ds_) and the sediment was weighted (w_ss_). Measurements were carried out in triplicate.

(1) $$\mathrm{WAI}\;\left(\frac{g}{g}\right)=\frac{w_{\mathrm{ss}}}{w_{0}},$$

(2) $$\mathrm{WSI}\;\left(g/100\;g\right)=\frac{w_{\mathrm{ds}}}{w_{0}}\times 100,$$

(3) $$\mathrm{SP}\;\left(g/g\right)=\frac{w_{\mathrm{ss}}}{w_{0}-w_{\mathrm{ds}}}$$

#### 2.7.2. Bulk Density

Bulk density of flours was determined using the method of Kaushal et al. [39]. Two grams of extruded and milled sample was filled into a 10 mL graduated cylinder and the volume was determined. Bulk density was expressed as weight of sample per unit volume of sample (g mL^−1^). Measurements were carried out in triplicate.

#### 2.7.3. Pasting Properties

Rapid Visco Analyzer, RVA-4 (Perten Instruments, Newport Scientific, Warriedwood, Australia) was used to determine the pasting properties of the flours. Before analysis, samples were sieved with a mesh size of 250 µm. Three grams of flour and 25 g of distilled water were dispensed, carefully, in RVA canisters, adjusting the weight of sample and water according to the moisture of each sample. After this, the paddle was placed inside the canister. Depending on the samples (M or E), different profiles were run. In the case of flours without extrusion (M), samples were equilibrated at 50 °C for 1 min, heated to 95 °C, held for 2.5 min, then cooled to 50 °C. The sample is again kept at 50 °C for 2 min. The speed of the paddle remains constant throughout the analysis except for the first 10 s during which the speed increases to disperse the sample. For extruded flours (E), samples were equilibrated at 25 °C for 2 min, heated to 95 °C for 3 min and then cooled to 25 °C, keeping that temperature for 5 min. As in the other method, there was an initial speed of 960 rpm for 10 s and then speed was kept constant the rest of the test. Obtained parameters were peak viscosity (PV), trough viscosity (TV), breakdown viscosity (BDV), final viscosity (FV), setback (SB) and pasting temperature (PT). For extruded flours, some other parameters are obtained: cold peak, raw peak, hold, peak time and cold peak area. All viscosity measurements were carried out in triplicate. Supplementary Figure S1 shows graphically the parameters evaluated.

### 2.8. Scanning Electron Microscopy (SEM)

Scanning electron microscopy (SEM) was carried out using a scanning electronic microscope (FEI QUANTA 200). For all flour samples, images were taken using two standard magnifications, 100 and 1000. Voltages between 10 and 12.5 kV have been used depending on the detector and the topography of the sample and the spot sizes (between 6 and 7) suitable for it.

### 2.9. Extract Preparation

For the extraction, one gram of flour (<0.5 mm) sample was resuspended in 10 mL of methanol:water (1:1, *v:v*; acidified to pH 2 with 0.1M HCl) in a controlled-temperature orbital shaker (25 °C, 250 rpm, 1 h). After centrifugation (2.057× *g* 10 min), the supernatant was collected and filtered (Whatman paper nº1). The residue was then re-extracted with 10 mL of methanol. The combined fractions were adjusted to 25 mL with extracting solvent added through the filter. Extract aliquots were stored at −80 °C until further analysis.

### 2.10. Total Phenol (TP)

TP content was measured using the Folin–Ciocalteu method as described by Slinkard and Singleton, with modifications [40]. The absorbance values were measured at 765 nm with a microplate reader (Fluostar Omega, BMG Ortenberg, Germany). Results were expressed as mg gallic acid equivalents (GAE) 100 g^−1^ of sample using a calibration curve with gallic acid as standard (9.8–70 µM). Samples were evaluated in duplicate.

### 2.11. Antioxidant Activity (AA)

AA was measured on extracts using 2,2-diphenyl-1-picrylhydrazyl radical (DPPH^•^), and 2,2′-azinobis-(3-ethylbenzothiazoline-6-sulfonate (ABTS^•+^), oxygen radical absorbance capacity (ORAC) and ferric reducing ability potential (FRAP) assays. DPPH^•^ and ABTS^•+^ modified methods were applied on solid samples without previous extraction as quencher methodologies (Q-DPPH^•^ and Q-ABTS·), in order to evaluate the total antioxidant activity of whole integrated samples. Samples were evaluated in duplicate.

#### 2.11.1. DPPH^•^ Radical Scavenging Activity and Q-DPPH^•^ Radical Scavenging Activity

The extract-based DPPH^•^ assay was carried out as described by Brand-Williams et al. [41], with modifications. A 120 µM DPPH^•^ working solution in pure methanol was prepared. In a 96-well microplate, a volume of 25 µL of extracts was mixed with 100 µL of milliQ water and 125 µL of DPPH^•^ working solution. The decay absorbance at 525 nm was recorded over 30 min with a microplate reader (Fluostar Omega, BMG, Ortenberg, Germany). Different solutions of Trolox (7.5–240 µM) were evaluated to perform a calibration curve. Results were expressed as mg Trolox equivalents (TE) 100 g^−1^ sample.

The solid sample-based Q-DPPH^•^ method was assayed following the procedure by Serpen et al. [42], with modifications. Ten milligrams of solid samples (<300 µm) were mixed with 30 mL of DPPH^•^ working solution (60 µM) prepared in methanol. After incubation at 700 rpm for 30 min (Thermomixer Compact, Eppendorf, AG, Hamburg, Germany), samples were centrifuged at 14,000× *g* for 2 min and the absorbance was measured at 515 nm. Results were expressed as mg Trolox equivalents (TE) 100 g^−1^ sample.

#### 2.11.2. Oxygen Radical Absorbance Capacity (ORAC)

The method was based on a previously reported method by Ou et al. [43], with some modifications. Standard curve of Trolox (7.5–240 mM) and samples were diluted in phosphate buffer (10 mM, pH 7.4). Fluorescence was monitored over 150 min with a microplate reader (Fluostar Omega, BMG, Ortenberg, Germany), using 485 nm excitation and 520 nm emission filters. Results were calculated using the areas under the fluorescein decay curves, between the blank and the sample, and expressed as mg Trolox equivalents (TE) 100 g^−1^ sample.

#### 2.11.3. ABTS^•+^ Radical Cation Scavenging Activity and Q-ABTS^•+^ Radical Cation Scavenging Activity

ABTS^•+^ was measured following the method first described by Miller and Rice-Evans [44], as modified by Martin-Diana et al. [45]. The absorbance was measured at 730 nm. Results were expressed as mg Trolox equivalents (TE) 100 g^−1^ sample.

The Q-ABTS^•+^ method described by Serpen et al. [42], as modified in Martin-Diana et al. [45], was applied to evaluate the direct antioxidant capacity of samples. Ten milligram of sample was mixed with 30 mL ABTS^•+^ working solution. A volume of 3 mL methanol:water (50:50 *v:v*) was added to the sample assays to equal the final volume present in the calibration curve run. A calibration curve with Trolox as standard (7.5–240 µM) was used. After 30 min of incubation in darkness, the decay in absorbance was measured at 730 nm. Results were expressed as mg Trolox equivalents (TE) 100 g^−1^ sample.

#### 2.11.4. Ferric Reducing Antioxidant Power (FRAP)

FRAP assay was performed following the protocol reported by Pereira et al. [46] Absorbance at 700 nm was recorded. FeSO_4_·7H_2_O was used as the standard (4.0–2.2 mM). The results were expressed as mmol Fe Equivalents (Eq.) 100 g^−1^ sample.

### 2.12. Glycaemic Index (GI)

For the evaluation of glycaemic index (GI) in non-extruded and extruded samples, first, the content of available starch was measured using the total starch assay kit of Megazyme (K-TSTA). Afterwards, the in vitro starch hydrolysis rate was measured as described by Gularte and Rosell [47], with some modifications. Samples with 50 mg of available starch were dissolved in tris-maleate buffer (0.1 M, pH = 6, 2 mL) and then 2 mL enzymatic solution containing porcine pancreatic α-amylase (460 U mL^−1^) and amyloglucosidase (6.6 U mL^−1^) was added. Aliquots were incubated for 150 min, and then were kept in boiling water for 5 min to stop the enzymatic reaction and cooled in ice. Then, a volume of 150 µL of absolute ethanol was added and the sample was centrifuged (4 °C, 10,000× *g* for 5 min). The pellet was washed with 150 µL ethanol:water (1:1, *v:v*) and the supernatants were pooled together and stored at 4 °C for the following colorimetric analysis of reducing sugars using the GOPOD kit (Megazyme, Bray, Ireland). GI values were expressed from hydrolysis index (HI) values, as proposed by Granfeldt [48].

### 2.13. Statistical Analysis

All data were expressed as the mean ± standard deviation. Analysis of variance (ANOVA) and post hoc Duncan’s test was used to identify the differences between mean values. The statistical analyses were used with a Statgraphics Centurion XVI^®^ (StatPoint Technologies, Inc., Warrenton, VA, USA).

## 3. Results and discussion

### 3.1. Proximal Analysis

Table 2 shows the proximal analysis of all the native (non-extruded- rice four, white and brown teff) and blend extruded flours (Table 1) analysed. The rice flour (RF) showed a content in ash significantly (*p* ≤ 0.05) lower than teff flours (WTF and BTF). Ash in RF had values of 1.50 g 100 g^−1^, higher than the values reported in other polished white rice by other authors (0.27–0.54 g 100 g^−1^) [49,50,51], and lower than that found in brown rice [52] (1.99–2.2 g 100 g^−1^). Teff ash content ranged from 2.50 to 3.44 g 100 g^−1^ with significant differences (*p* ≤ 0.05) between both teff types; differences among teff types were also reported by other authors previously [17] although values, regardless of type of teff (WTF and/or BTF), were in all the cases higher to those observed in this study [53]. The ash content has a positive correlation with the mineral content of the grains, and differences observed can be associated to dehulling practice, genetic variation, implemented agronomic practices or soil characteristics, among other factors. High ash levels of teff harvested in Ethiopia, as compared to those from Europe, are due to higher iron and other minerals presence in Ethiopian soils [53].

The content of ashes was evaluated after extrusion; the results showed that the ash was reduced by 45% in the extruded RF (E1), decreasing the ash loss percentage when rice was combined with 50% teff, where reductions did not exceed 33% (E5, E6 and E7). Minerals have been considered very stable during extrusion [54,55]. However, the authors have previously observed a decrease in ash content after extrusion in pulses [56]. This behaviour could be associated to the interaction of minerals with fibre and protein during extrusion [57], which would explain the limited ash loss from teff, as compared to rice, after the higher content in protein and TDF present in the cereal (WTF and BTF).

The moisture content ranged from 10.01 g 100 g^−1^ to 11.74 g 100 g^−1^ for RF and teff flours before extrusion. A significant reduction of the moisture content was observed after extrusion, with values equal or below 7.90 in all cases (Table 2), values which ensure the safe storage of the flours and their stability [58].

Fat also was evaluated before and after the extrusion; the levels of fat were lower in RF than those in WTF-BTF, with significantly higher values in white (*p* ≤ 0.05) compared to brown. The extrusion did not produce any effect in total fat values, probably due to the low content of fat in the sample. On matrices with high fat content, a fat content decreased from 42% to 30% after extrusion was observed, as in the case of seed flake processing [59], which was attributed to disruption of cell walls, increasing migration of oil outside the food matrix [60].

Total dietary fibre (TDF) of samples with RF and teff flour mixes significantly (*p* < 0.05) increased after extrusion (16 to 100% increase from native flours), while TDF of RF increase (10%) was not significant. Extruded flours with 50% teff resulted in significantly higher TDF values than those of containing 25% teff flour. Many studies have shown significant effects of extrusion on fibre composition in many matrices [61,62], resulting in a reduction in the ratio of insoluble to soluble fibre; but few works have reported increases in total fibre content overall [63,64], as it occurred in this work. High shear–temperature combination of different mechanism by which extrusion could cause carbohydrate–lipid–protein complexes, lead to the formation of resistant starch [65].

The crude protein was analysed before and after extrusion; the native flours had similar values to those found by the authors in previous works with rice [56] and teff flours [17], and in the range of those reported by other authors [66]. Extrusion produced a significant reduction of crude protein content (Table 2); the temperature and shear forces applied during the thermal processing might have favoured the unfolding of the protein structures and enhanced their digestibility [67].

Carbohydrate content was similar in RF to values reported by other authors [56], and in teff flours, values were slightly higher (81.67 to 81.97 g 100 g^−1^) than those reported by other authors, where teff carbohydrates reached values of 73.1 and 73.6 g 100 g^−1^ [68]. A significant reduction of carbohydrates was found, similarly to other authors who have previously reported decreases in carbohydrates after extrusion of cereal matrices [69]. This is probably due to partial hydrolysis of starch, as it has been previously reported [70].

### 3.2. Colorimetric Analysis

**Figure 2:**
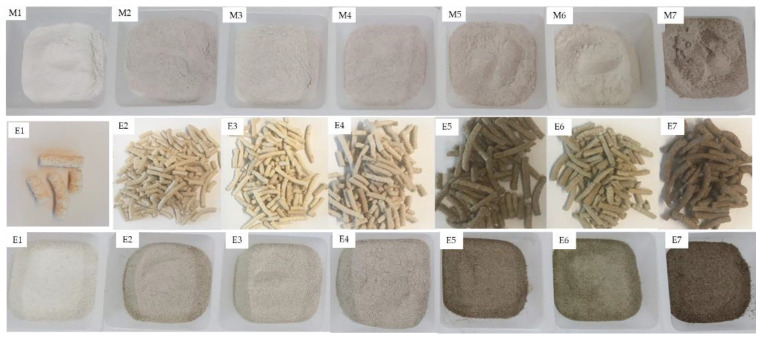
Image of blend mixtures (M1–M7) before extrusion and E1–E7: Extruded pellets (middle row) and flours produced from extruded (bottom row). M1/E1(100RF), M2/E2 (12.5WTF/12.5 BTF/75RF), M3/E3 (25WTF/75RF), M4/E4 (25BTF/75RF), M5/E5 (25WTF/25 BTF/50RF), M6/E6 (50WTF/50RF), M7/E7 (50BTF/50RF).

Colour was evaluated in the native and extruded flours (Table 3, Figure 2). The extrudates flours were darker in colour compared to their native blends. Luminosity values of the extruded were reduced from 119.34–94.80 to 114.55 to 72.17, being lower in E7 (72.17). Meanwhile the reduction in RF (E1) was 4.20%, and in the blend formulas the reduction reached 23.40% (E7). Similar findings have been reported by other authors such as Leonel et al. [71] and Bhattacharya et al. [72], and this reduction in luminosity might be associated to the formation of brown pigments through non-enzymatic Maillard reactions between proteins and reducing sugars that occur during the product processing [73].

The a* (green to red) colour parameter significantly changed in some of the blends, although a not clear trend was observed; meanwhile, b* (blue to yellow) showed significant increase in all the formulas, indicating a higher yellow component in the colour of the extruded samples.

The results agreed with the results reported by Paes and Maga [74] where an increase was observed in extruded flours. Contrary to the effect observed with L* values, which were modified to a higher degree with increasing teff concentration (50% teff flours), higher changes in b* values after extrusion were observed in RF and blends with lower percentage of teff (25%). These colour (Figure 2) changes after extrusion significantly modified the aspect to different degrees of the extruded flours

### 3.3. Functional Properties

#### 3.3.1. Hydration Properties

Extrusion has been reported to affect hydration properties of flours through increasing starch damage [75], which subsequently increases the water holding capacity of cereal flours and the ability to enhance water absorption and starch digestibility during the dough making, as a result of high levels of starch exposed for hydration and enzymatic action [76]. Results of hydration properties of teff and rice flours are shown in Table 4 and Table 5.

WAI was evaluated, since it measures the volume occupied by the gelatinised starch after swelling in excess water, reflecting the integrity of starch in aqueous dispersion [39] and its degree of degradation of starch [28]. Extrusion produced a significant (*p* ≤ 0.05) increase of water absorption index (WAI) in all samples, with the exception of RF. This increase was higher in blend flours where the concentration of teff was 50% (E5–E7), as compared to 25% teff mixtures. WAI varies with the gelatinisation degree of starch, which is changed during the extrusion process. A higher gelatinisation degree of the starch means a greater number of available hydroxyl groups of this molecule to form hydrogen bonds with water and, in consequence, increased WAI values [77]. Along with starch gelatinisation, protein denaturation and solubilisation of insoluble fibre fraction, which occur during extrusion, could also be responsible for increasing WAI value of extrudates [78].

Water solubility index (WSI) and swelling power (SP) increased after extrusion in all cases (E1–E7). This also reflects starch gelatinisation occurring during extrusion cooking. Higher WSI values in the extruded samples were obtained with increasing teff concentration, especially for those formulated with rice and brown teff (E4 and E7), while extruded 100% rice (E1) showed the lowest value (Table 4). The WSI reflects the degree of gelatinisation, dextrinisation, and solubilisation of the starch granules, by measuring the amount of the soluble components that were released from the starch after the extrusion, this behaviour was caused by an intense shear fragmentation of the starch granules during the extrusion process [79]. The native flours (M1–M7) also revealed higher WSI values for those formulated with Teff, as compared with rice only native flour (M1), which resulted in the lowest WSI value of all evaluated samples.

Swelling Power (SP) showed same behaviour than WSI Teff flours showed higher SP in native form and after extrusion (Table 4). Moreover, BTF showed higher SP than WTF. Which would indicate the higher ability to solubilise polysaccharides in brown compared to white teff.

WHC determine the water retained by the sample without being subjected to any stress [80]. Both WHC and SV are significantly modified in all the flours after extrusion.

WAC is defined as the ability of the flour to absorb water. OAC determines the ability of flour proteins to physically bind fat by capillary attraction [39]. This is an important parameter because fat acts as flavour retainer, increasing the mouthfeel [39,80]. Higher oil absorption capacity means higher flavour retention, improvement of palatability and extension of shelf life, which are interesting for food products, mainly baked and meat [80]. OAC increased after extrusion of 100% rice flour, but no significant changes were observed in rice-teff flours before and after extrusion, while WAC increased significantly after extrusion in all flour blends.

An increase was observed in the different hydration properties of extruded flours in relation to native ones, denoting the effect of extrusion technology on functional and technological properties of rice and teff flours. OAC was the only parameter not affected by extrusion, besides 100% rice (M1–E1). All these results reveal the effect of technology extrusion on starch gelatinisation and protein denaturalisation, which affects flour properties [21].

#### 3.3.2. Bulk Density

The bulk density (BD) results of the different samples are shown in Table 6. Bulk density did not show significant differences (*p* < 0.05) among formulas (M1 to M7); however, extrusion produced a significant decrease in BD in all cases. On the contrary, Meuser and Wiedmann [81] observed an increase in bulk density with increasing extrusion temperatures, which is explained by melting and liquefaction of sugar. BD values ranged from 812.7 to 842.0 before extrusion and from 691.6 to 754.5 after extrusion. Higher BD values can be due to high fibre contents in formulas where teff has been incorporated, which contributes to the increase in the bulk density of the flour. Different authors have explained this phenomenon due to the interaction of fibre and protein-rich materials added to starchy matrices [81,82]. However, other authors have reported that at high temperature the vapor pressure of the free moisture increases, which results in higher moisture flashing and puffing up rates on exit from the die [59,83], effect that would lead to a decreased bulk density, as it was observed in our results.

#### 3.3.3. Pasting Properties

Results of the analysis on pasting properties of native blend flours are shown in Table 7. Significant differences were observed in all pasting parameters associated to the inclusion of teff in the rice matrix. Pasting, through, breakdown, final and setback viscosities were significantly reduced with increasing teff concentrations. Differences in the viscosity patterns among starches have been explained by starch polymorphism (e.g., A-type in case of wheat and waxy, B-type for potato), properties that are dependent on the microstructure of native starch granules (lamellar organisation and branch-chain length of amylopectin). Rice and teff starch granules have been reported to be of similar size (2–6 µm), shape (polygonal) and crystallinity [14,84,85]. Therefore, differences may be due to other factors, such as protein–starch and fat–starch interactions or fibre content. Increasing protein has been observed to affect viscosity of oat starch, and also affect the pasting temperature [86], as it occurred in samples M5 and M6, where starch gelatinisation required a higher energy input. Similarly, fat–starch interactions have been observed to increase peak, breakdown and final viscosity of starchy matrices [87]. The reduced viscosity parameters observed with increasing teff percentage in the mixtures reflect higher stability and retrogradation, while decreasing teff percentage in the mixtures resulted in flours with improved swelling capacity and thermal stability. Increased pasting temperature with increasing teff content indicates higher resistance of teff starch to water absorption, and probably better suitability for standing high-temperature processes.

After extrusion, pasting properties were modified (Table 8). All extruded samples showed significant cold peak areas, indicating the ability of their starch to absorb water with no thermal energy input, except for the 50% white teff extruded sample (E6). Cold peak values observed are consistent with the higher WAC values obtained for extruded samples, as compared to native, non-extruded flours (Table 7). Viscosity values (breakdown, final and setback) were significantly lower than those of the non-extruded samples, indicating more stable starches. These changes, along with increased holding viscosity, were observed as well with increasing teff content in the mixtures. Individual rheological behaviour of each sample appears in Supplementary Figures S2 and S3.

#### 3.3.4. Microstructure

**Figure 3:**
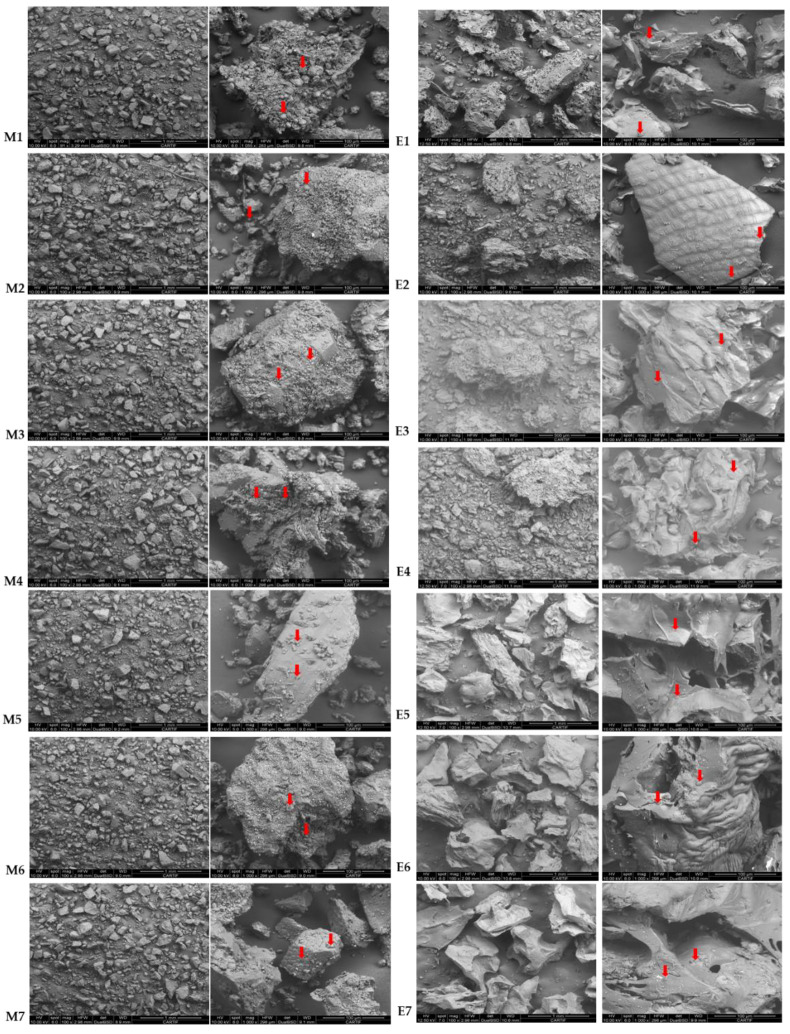
Scanning electron microscope (SEM) images of Native (M1–M7) and extruded (E1–E7) rice and blend flours. Arrows indicate starch granules. (M1–M7). M1/E1(100RF), M2/E2 (12.5WTF/12.5 BTF/75RF), M3/E3 (25WTF/75RF), M4/E4 (25BTF/75RF), M5/E5 (25WTF/25 BTF/50RF), M6/E6 (50WTF/50RF), M7/E7 (50BTF/50RF).

Microstructure was analysed in all the samples before and after the extrusion (M1–M7 and E1 and E7). The starch granules are clearly identified in the native flours (M1–M7), while loss of starch granule shape and structure in the extruded flours (E1–E7) is revealed from microstructure images (Figure 3), indicating starch gelatinisation. The surfaces of intact, broken and extruded rice showed a mud-like superficial structure, which indicated that the starch on the surface was fully cooked. The same effect was also observed in the images with teff blends regardless of the concentration and type of teff used.

After extrusion, it can be observed a smaller average particle size in samples E1–E4 than that observed in samples where teff was used at 50% (E5–E7), which apparently show more plasticised structures, which can be observed at both magnification micrographs.

### 3.4. Total Phenols (TPs)

**Figure 4:**
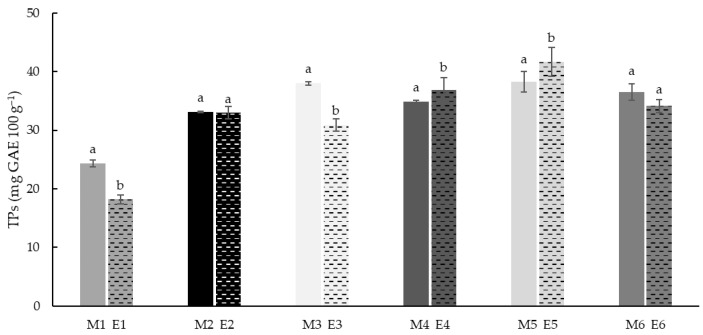
Total phenols (TPs) of native (M1–M7) and extruded (E1–E7) blend flour samples. The values are expressed as mg gallic acid equivalent (GAE) 100 g^−1^ of dry matter. Different letters indicate significant differences (*p* < 0.05) in TPs between non-extruded (M) and extruded (E) flours, for each blend formulation.

The phenolic total content was analysed in all the native and extruded samples. The results showed (Figure 4) a significant (*p* < 0.05) increase of phenol content with increasing teff concentration. Rice flours (M1 and E1) had the lowest TP values (24.3 and 18.2 mg GAE 100 g^−1^ respectively). These results agreed with the previous studies reported [56,88], where it was observed that rice flours after extrusion significantly reduced (*p* ≤ 0.05) its phenolic content, which may demonstrate that the phenolic compounds in rice flours are more thermally sensitive than in other flours.

The samples with 25% Teff (M2–M4, E2–E4) had higher phenolic content (30.8 to 38.0 mg GAE 100 g^−1^). Finally, samples with 50% teff showed the highest range of TP values (34.1 to 42.1 mg GAE 100 g^−1^). This agrees with previous results reported by the authors [17] where native white and brown teff flours showed three-fold concentrations of phenols in rice flour. The extrusion decreased TP content in rice flour and those samples with 25 and 50% of white teff (E1, E3 and E6).

Some authors [89,90] reported higher soluble phenolic content in white tef varieties, while brown varieties showed higher bound and total phenolic content. As previously reported by other authors [91], soluble-free and soluble-conjugated phenolic acids were transformed into insoluble-bound phenolic acids after extrusion, which might explain the reduction in TP in white teff-containing samples, as compared to those containing brown teff, where TP levels were maintained after extrusion. On the other hand, the increment observed in the case of brown teff after extrusion could be partially associated to the breakdown of ester bonds between phenolics and cell walls or components of complex structures such as cellulose, lignin and proteins. A higher amount of bound phenolics in brown teff was found as compared to white teff, where the soluble phenolic fraction was majorly described [92]. Since the samples were subjected to high temperature, the increment observed also could be associated to Maillard reaction products which act as reducing agents with Folin Ciocalteau reagent in the TP analysis [93].

### 3.5. Total Antioxidant Capacity (TAC)

Analysis were evaluated through the use of different markers (DPPH^•^, ABTS^•+^, ORAC and FRAP) in order to get insight into the effect of different antioxidants against free radical. The extrusion process produced a reduction in DPPH^•^ antioxidant activity in rice (226.25 to 212.43 mg TE 100 g^−1^), although this difference was not significant. Meanwhile, in the case of teff blends, the extrusion enhanced significantly (*p* < 0.05) the DPPH^•^ scavenging activity, with maximum activity observed blends with 50% teff concentration (E5–E7). Moreover, formulations with brown teff and mixed brown and white varieties showed higher antioxidant activity than samples with white teff. The higher antioxidant activity observed in BTF could be explained through a higher TP release from the bound phenolic fraction [92].

The ABTS^•+^ radical scavenging activity showed differences associated to extrusion process in rice (433.02 and 274.19 mg TE 100 g^−1^, before and after extrusion respectively). No differences were observed in teff blend flours after extrusion, maintaining similar values to those of non-extruded native blends.

The DPPH^•^ method yielded lower TAC values than the ABTS assay. DPPH^•^ is characterised by a lower sensitivity than ABTS^•+^ assay [94]. ABTS^•+^ radical reacts with antioxidants with a higher polarity range than DPPH^•^ radical. It is probable that the differences observed between the DPPH^•^ and ABTS were due to the presence of antioxidants in the extracts with intermediate character between polar and non-polar, as for example the presence of free α-linolenic, a fatty acid with reported antioxidant activity that is relatively abundant in teff, as compared to other cereals [95,96].

**Figure 5:**
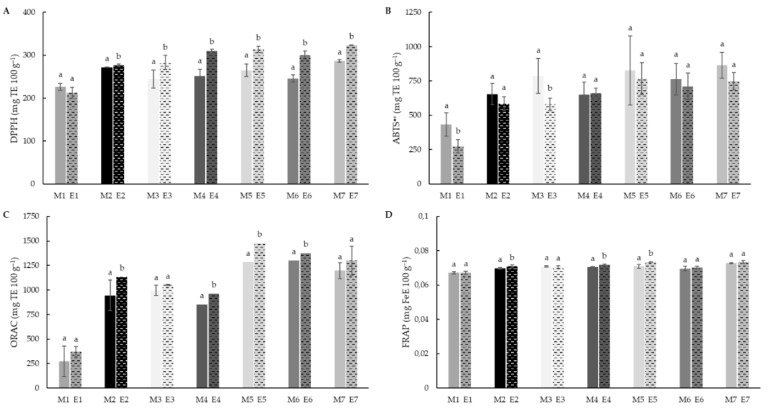
DPPH (**A**), ABTS^•+^ (**B**), ORAC (**C**) and FRAP (**D**) values of native (M1–M7) and extruded (E1–E7) blend flour samples. The DPPH, ABTS and ORAC values are expressed as mg Trolox equivalent (TE) 100 g^−1^ of dry matter, and FRAP values as mg ferric reduced equivalent (FeE) 100 g^−1^ of dry matter. Different letters indicate significant differences (*p* < 0.05) between non-extruded (M) and extruded (E) flours, for each blend formulation. M1/E1(100RF), M2/E2 (12.5WTF/12.5 BTF/75RF), M3/E3 (25WTF/75RF), M4/E4 (25BTF/75RF), M5/E5 (25WTF/25 BTF/50RF), M6/E6 (50WTF/50RF), M7/E7 (50BTF/50RF).

ORAC showed an increase in activity after the extrusion, this behaviour being significant in teff-containing samples (Figure 5C). Increasing teff concentrations were also reflected in significant (*p* < 0.05) increases of ORAC values, a trend observed after DPPH^•^ and ABTS^•+^ values although not significantly. On the other hand, the reducing potential (FRAP, Figure 5D) was neither affected by the addition of teff flour to the formulation, nor by the extrusion process.

**Figure 6:**
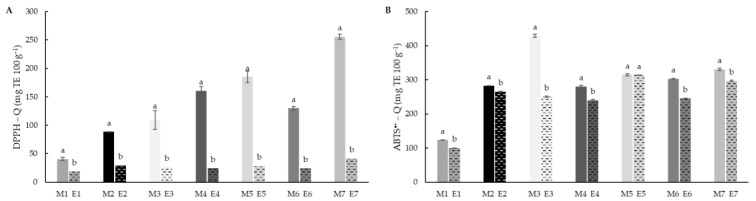
Quencher DPPH (**A**) and Quencher ABTS^•+^ (**B**) values of native (M1–M7) and extruded (E1–E7) blend flour samples. Results were expressed as mg Trolox equivalent (TE) 100 g^−1^ of dry matter. Different letters indicate significant differences (*p* < 0.05) between non-extruded (M) and extruded (E) flours, for each blend formulation. M1/E1 (100RF), M2/E2 (12.5WTF/12.5 BTF/75RF), M3/E3 (25WTF/75RF), M4/E4 (25BTF/75RF), M5/E5 (25WTF/25 BTF/50RF), M6/E6 (50WTF/50RF), M7/E7 (50BTF/50RF).

Antioxidant activities of the flour samples were evaluated before and after extrusion using direct methods (Figure 6). Q-DPPH^•^ and QABTS^•+^ showed similar behaviour and values, probably because the evaluation was carried out directly on the sample instead of the extracts and the differences associated with the polarity in this study are reduced. In all the cases, significant (*p* < 0.05) reduction after extrusion was observed, especially in DPPH^•^ scavenging activity. In the case of the ABTS^•+^, the decrease in antioxidant ability was higher in extruded compared to DPPH^•^ and extractive methods, the difference can also be due to the aggregation of fats and protein and fibre during extrusion [65] which can reduce the ability to scavenge the ABTS^•+^ radical. Although rice had significant lower values in direct methods, no differences were observed in the case of white and brown teff.

### 3.6. Gyceamic Index (GI)

**Figure 7:**
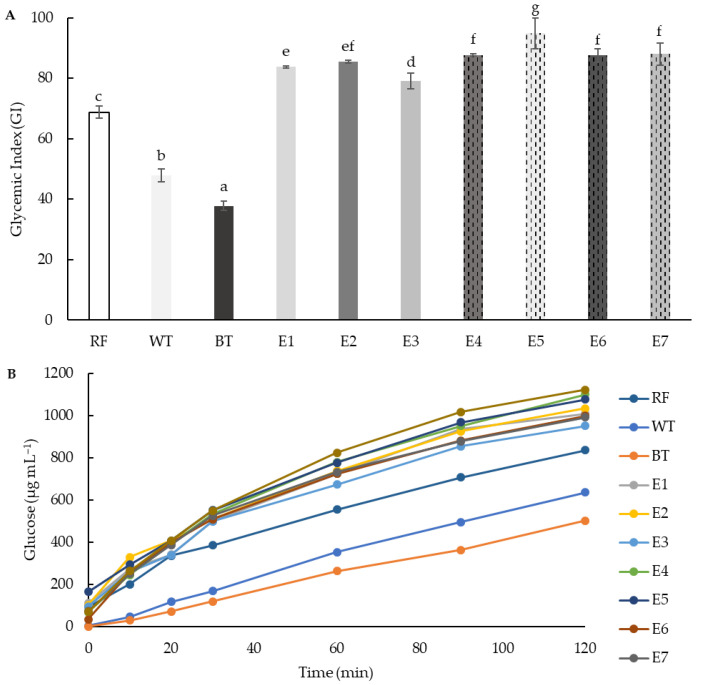
(**A**) Glycaemic index (GI) and (**B**) glucose kinetics consumption (μg mL^−1^) of native rice (RF), white teff (WT) and brown teff (BT) flours, and extruded blend flours (E1–E7). Different letters indicate significant differences (*p* < 0.05) between samples. M1/E1 (100RF), M2/E2 (12.5WTF/12.5 BTF/75RF), M3/E3 (25WTF/75RF), M4/E4 (25BTF/75RF), M5/E5 (25WTF/25 BTF/50RF), M6/E6 (50WTF/50RF), M7/E7 (50BTF/50RF).

Starch hydrolysis kinetics and estimated GI were evaluated in all extrusion samples and in native rice and teff (white and brown) flours. The hydrolysis kinetics of the starch in the extruded samples were similar to those of the white bread used as control (GI = 100), although with lower levels of released glucose in samples containing 25% teff than those with 50% teff (Figure 7B). Native teff flours showed lower hydrolysis kinetics and GI than extruded samples. The GI of native flours has been closely related to the crystallinity structure of the starch [97]. Moreover, the higher GI of white variety could be due to its higher protein content [98]. The extrusion increased the glycaemic index of the extruded samples to levels close to 100 (Figure 7A), which can be considered as high GI values [99]. Increasing concentrations of teff in the mixtures resulted in significantly higher glycaemic indexes.

Observed increases of GI in extruded products have been previously explained through gelatinisation and fragmentation of starch, and also due to structural and conformational changes in the protein fraction, leading to overall increased digestibility [100,101]. Starch is easily available to enzymatic hydrolysis. On the other hand, some authors have pointed out factors that can lead to a reduction in GI, such as cross-link (indigestible linkages) between small amylose and amylopectin fragments, and between amylose and lipids [102], and therefore not a complete consensus exists on the effect of extrusion on the glycaemic properties of starchy foods.

## 4. Conclusions

The extrusion produced in all the blends, and especially in those with higher concentration of teff, an increased ability to absorb water, resulting in higher determined values of WAI and SP. Pasting properties improved after extrusion and with increasing teff concentration, obtaining starches with higher shearing and heating stabilities. These properties of teff blends pasting properties were reflected in their microstructure (SEM), where a higher average particle size and apparently more plasticised structures with increasing teff concentrations were observed. Furthermore, a significant effect increasing the glycaemic index with increasing teff concentration was observed, results consistent with a better-packed starch structure, as compared to that of rice flour.

Along with these improvements through extrusion in teff containing rice flours, increased nutritional value (fibre content), phenolic compound solubility and antioxidant activities were also observed, especially when brown teff was used in the flour blend formulation. These results show the potential use of teff in extrusion processing for providing technological solutions which require flours with higher starch stability than that provided by rice starch, a commonly used flour in gluten-free products. Teff also may increase the nutritional and bioactive value of the final products.

Meanwhile native teff showed a drawback for the incorporation in formulas due to the poor techno-functional properties. The use of extrusion will facilitate the possibility to use teff as a GF flour for a wide range of products such as thickening ingredients, which have a potential to be used in food, especially in preparation of modified flours with better gelling properties.

## 5. Patents

No patents have been described associated to this study.

## Figures and Tables

**Table 1:** Blend flour formulations in percentage (%). *Abbreviations: RF: rice flour, WTF: white teff flour, BTF: brown teff flour*.

Blend Fours	WTF	BTF	RF
1	0	0	100
2	12.5	12.5	75
3	25	0	75
4	0	25	75
5	25	25	50
6	50	0	50
7	0	50	50

**Table 2:** Proximal analysis of native and extruded blend flours. The values are expressed as g 100 g^−1^ of dry matter (d.m.).

Blends (g 100 g^−1^)	Ash (g 100 g^−1^)	Fat (g 100 g^−1^)	TDF (g 100 g^−1^)	Moisture (g 100 g^−1^)	Proteins (g 100 g^−1^)	Carbohydrates (g 100 g^−1^)
RF	1.50 ± 0.00 ^c^	1.19 ± 0.02 ^a^	1.65 ± 0.01 ^a^	10.01 ± 0.12 ^d^	8.48 ± 0.01 ^a^	88.83 ± 0.03 ^e^
WTF	3.44 ± 0.03 ^f^	2.76 ± 0.08 ^e^	4.54 ± 0.15 ^c^	11.13 ± 0.13 ^e^	12.13 ± 0.03 ^d^	81.67 ± 0.07 ^cd^
BTF	2.50 ± 0.02 ^e^	2,24 ± 0.20 ^d^	4.45 ± 0.76 ^c^	11.74 ± 0.56 ^e^	13.28 ± 0.13 ^e^	81.98 ± 0.35 ^d^
E1 (100 RF)	0.82 ± 0.05 ^a^	1.28 ± 0.07 ^a^	1.85 ± 0.23 ^a^	7.90 ± 0.45 ^c^	8.35 ± 0.47 ^a^	79.80 ± 1.27 ^bcd^
E2 (12.5 WTF/12.5 BTF/75 RF)	1.16 ± 0.07 ^b^	1.79 ± 0.10 ^bc^	4.08 ± 0.52 ^bc^	6.85 ± 0.39 ^b^	9.26 ± 0.52 ^ab^	76.85 ± 1.60 ^ab^
E3 (25 WTF/75 RF)	1.14 ± 0.06 ^b^	1.65 ± 0.09 ^b^	3.50 ± 0.45 ^b^	5.82 ± 0.33 ^a^	9.16 ± 0.52 ^ab^	78.72 ± 1.45 ^bc^
E4 (25 BTF/75 RF)	1.09 ± 0.06 ^b^	1.72 ± 0.10 ^b^	3.93 ± 0.50 ^b^	5.81 ± 0.33 ^a^	9.49 ± 0.54 ^bc^	77.96 ± 1.52 ^b^
E5 (25 WTF/25 BTF/50 RF)	1.54 ± 0.09 ^cd^	2.02 ± 0.11 ^cd^	5.76 ± 0.73 ^d^	6.17 ± 0.35 ^ab^	10.06 ± 0.57 ^bc^	74.44 ± 1.85 ^a^
E6 (50 WTF/50 RF)	1.67 ± 0.09 ^d^	1.60 ± 0.09 ^b^	6.22 ± 0.79 ^d^	6.73 ± 0.38 ^b^	9.59 ± 0.54 ^bc^	74.20 ± 1.90 ^a^
E7 (50 BTF/50 RF)	1.63 ± 0.09 ^cd^	2.15 ± 0.12 ^d^	5.52 ± 0.70 ^d^	5.75 ± 0.33 ^a^	10.34 ± 0.59 ^c^	74.61 ± 1.83 ^a^

Different letters indicate significant differences (*p* < 0.05) within parameters. Abbreviations: RF: rice flour, WTF: white teff flour, BTF: brown teff flour, E1–E7: extruded blend flours. TDF: total dietary fibre. E1(100RF), E2 (12.5WTF/12.5 BTF/75RF), E3 (25WTF/75RF), E4 (25BTF/75RF), E5 (25WTF/25 BTF/50RF), E6 (50WTF/50RF), E7 (50BTF/50RF).

**Table 3:** Colorimetric analysis (CIE L* a* b*) of different blend flours (1–7).

	L*		a*		b*	
Blends (g 100 g^−1^)	M	E	M	E	M	E
1 (100RF)	119.34 ± 0.27 ^b^	114.55 ± 0.64 ^a^	−0.20 ± 0.11 ^a^	−0.16 ± 0.08 ^a^	8.58 ± 0.17 ^a^	12.44 ± 0.13 ^b^
2 (12.5WTF/12.5 BTF/75RF)	105.80 ± 0.12 ^b^	100.62 ± 0.38 ^a^	2.50 ± 0.03 ^a^	2.48 ± 0.09 ^a^	11.00 ± 0.09 ^a^	15.13 ± 0.39 ^b^
3 (25WTF/75RF)	109.70 ± 0.08 ^b^	102.35 ± 0.44 ^a^	1.30 ±0.05 ^a^	1.63 ± 0.05 ^b^	11.50 ± 0.07 ^a^	17.31 ± 0.09 ^b^
4 (25BTF/75RF)	102.40 ± 0.18 ^b^	98.94 ± 0.42 ^a^	3.50 ± 0.05 ^b^	3.08 ± 0.05 ^a^	10.90 ± 0.12 ^a^	13.33 ± 0.41 ^b^
5 (25WTF/25 BTF/50RF)	100.30 ± 0.15 ^b^	81.35 ± 2.70 ^a^	3.30 ± 0.08 ^a^	3.22 ± 0.39 ^a^	13.40 ± 0.18 ^a^	15.19 ± 0.50 ^b^
6 (50WTF/50RF)	107.50 ± 0.09 ^b^	87.01 ± 1.08 ^a^	1.20 ± 0.03 ^b^	0.73 ± 0.21 ^a^	15.30 ± 0.16 ^a^	15.98 ± 0.27 ^b^
7 (50BTF/50RF)	94.80 ± 0.29 ^b^	72.17 ± 1.79 ^a^	5.00 ± 0.08 ^a^	5.06 ± 0.38 ^a^	13.10 ± 0.17 ^a^	14.31 ± 0.69 ^b^

Different letters indicate significant differences (*p* < 0.05) between non-extruded (M) and extruded (E) flours, for each blend formulation and colour parameter. M1/E1(100RF), M2/E2 (12.5WTF/12.5 BTF/75RF), M3/E3 (25WTF/75RF), M4/E4 (25BTF/75RF), M5/E5 (25WTF/25 BTF/50RF), M6/E6 (50WTF/50RF), M7/E7 (50BTF/50RF).

**Table 4:** Hydration properties of flours expressed as water absorption index (WAI), water solubility index (WSI) and swelling power (SP).

.	WAI (g/g)		WSI (%)		SP (mL/g)	
Blends (g 100 g^−1^)	M	E	M	E	M	E
1 (100RF)	6.46 ± 0.56 ^a^	7.64 ± 0.06 ^a^	1.55 ± 1.07 ^a^	8.47 ± 0.49 ^b^	6.60 ± 0.55 ^a^	8.29 ± 0.03 ^b^
2 (12.5WTF/12.5BTF/75 RF)	5.43 ± 0.12 ^a^	6.31 ± 0.11 ^b^	3.36 ± 0.29 ^a^	11.35 ± 1.88 ^b^	5.59 ± 0.13 ^a^	7.08 ± 0.41 ^b^
3 (25WTF/75RF)	5.23 ± 0.23 ^a^	6.77 ± 0.14 ^b^	3.41 ± 1.05 ^a^	14.70 ± 0.41 ^b^	5.40 ± 0.20 ^a^	7.88 ± 0.19 ^b^
4 (25BTF/75RF)	5.59 ± 0.07 ^a^	6.23 ± 0.14 ^b^	2.22 ± 0.60 ^a^	16.59 ± 0.55 ^b^	5.71 ± 0.07 ^a^	7.41 ± 0.18 ^b^
5 (25WTF/25BTF/50RF)	5.17 ± 0.21 ^a^	6.84 ± 0.29 ^b^	2.52 ± 0.16 ^a^	21.02 ± 6.03 ^b^	5.29 ± 0.22 ^a^	8.58 ± 0.78 ^b^
6 (50WTF/50RF)	5.43 ± 0.12 ^a^	6.85 ± 0.24 ^b^	3.09 ± 0.25 ^a^	10.57 ± 0.77 ^b^	5.58 ± 0.13 ^a^	7.61 ± 0.21 ^b^
7 (50BTF/50RF)	5.29 ± 0.19 ^a^	7.37 ± 0.20 ^b^	2.82 ± 0.55 ^a^	25.11 ± 1.91 ^b^	5.43 ± 0.17 ^a^	9.69 ± 0.04 ^b^

Different letters indicate significant differences (*p* < 0.05) between non-extruded (M) and extruded (E) flours, for each blend formulation and hydration parameter. M1/E1(100RF), M2/E2 (12.5WTF/12.5 BTF/75RF), M3/E3 (25WTF/75RF), M4/E4 (25BTF/75RF), M5/E5 (25WTF/25 BTF/50RF), M6/E6 (50WTF/50RF), M7/E7 (50BTF/50RF).

**Table 5:** Hydration properties of flours expressed as water holding capacity index (WHC), swelling volume (SV), water activity (WAC), and oil absorption capacity (OAC).

	WHC (%)		SV (g/g)		WAC (g/g)		OAC (g/g)	
Blends (g 100 g^−1^)	M	E	M	E	M	E	M	E
1 (100RF)	2.11 ± 0.82 ^a^	7.88 ± 0.16 ^b^	2.93 ± 0.16 ^a^	10.81 ± 0.50 ^b^	2.64 ± 0.14 ^a^	6.80 ± 0.23 ^b^	1.10 ± 0.03 ^a^	2.05 ± 0.08 ^b^
2 (12.5WTF/12.5BTF/75 RF)	1.80 ± 0.03 ^a^	7.36 ± 0.64 ^b^	3.00 ± 0.16 ^a^	8.68 ± 0.25 ^b^	2.67 ± 0.07 ^a^	6.68 ± 0.09 ^b^	1.23 ± 0.15 ^a^	1.58 ± 0.20 ^a^
3 (25WTF/75RF)	1.75 ± 0.03 ^a^	6.60 ± 0.95 ^b^	3.06 ± 0.01 ^a^	8.13 ± 0.58 ^b^	2.55 ± 0.21 ^a^	6.60 ± 0.07 ^b^	1.50 ± 0.12 ^a^	1.58 ± 0.08 ^a^
4 (25BTF/75RF)	1.66 ± 0.03 ^a^	6.30 ± 1.25 ^b^	2.91 ± 0.16 ^a^	9.19 ±0.52 ^b^	2.60 ± 0.02 ^a^	6.87 ± 0.08 ^b^	1.63 ± 0.19 ^a^	1.70 ± 0.10 ^a^
5 (25WTF/25BTF/50 RF)	1.75 ± 0.03 ^a^	6.09 ± 0.39 ^b^	3.14 ± 0.16 ^a^	8.42 ± 0.01 ^b^	2.56 ± 0.04 ^a^	6.83 ± 0.42 ^b^	1.91 ± 0.11 ^a^	1.83 ± 0.08 ^a^
6 (50WTF/50RF)	1.77 ± 0.03 ^a^	7.54 ± 0.09 ^b^	3.16 ± 0.16 ^a^	9.43 ± 0.15 ^b^	2.52 ± 0.07 ^a^	6.59 ± 0.16 ^b^	1.68 ± 0.06 ^a^	1.67 ± 0.11 ^a^
7 (50BTF/50RF)	1.78 ± 0.02 ^a^	5.91 ± 0.83 ^b^	3.07 ± 0.01 ^a^	8.21 ±0.73 ^b^	2.62 ± 0.04 ^a^	6.93 ± 0.21 ^b^	1.56 ± 0.14 ^a^	1.64 ± 0.08 ^a^

Different letters indicate significant differences (*p* < 0.05) between non-extruded (M) and extruded (E) flours, for each blend formulation and hydration parameter. M1/E1(100RF), M2/E2 (12.5WTF/12.5 BTF/75RF), M3/E3 (25WTF/75RF), M4/E4 (25BTF/75RF), M5/E5 (25WTF/25 BTF/50RF), M6/E6 (50WTF/50RF), M7/E7 (50BTF/50RF).

**Table 6:** Bulk density of flours.

Blends (g 100 g^−1^)	M	E
1 (100RF)	812.7 ± 0.02 ^a^	691.6 ± 0.01 ^b^
2 (12.5WTF/12.5 BTF/75RF)	837.0 ± 0.02 ^a^	724.8 ± 0.01 ^b^
3 (25WTF/75RF)	842.0 ± 0.01 ^a^	710.4 ± 0.01 ^b^
4 (25BTF/75RF)	820.0 ± 0.00 ^a^	695.5 ± 0.01 ^b^
5 (25WTF/25 BTF/50RF)	817.4 ± 0.02 ^a^	715.6 ± 0.01 ^b^
6 (50WTF/50RF)	821.7 ± 0.00 ^a^	754.5 ± 0.01 ^b^
7 (50BTF/50RF)	819.9 ± 0.02 ^a^	691.6 ± 0.01 ^b^

Different letters indicate significant differences (*p* < 0.05) between non-extruded (M) and extruded (E) flours, for each blend formulation. M1/E1(100RF), M2/E2 (12.5WTF/12.5 BTF/75RF), M3/E3 (25WTF/75RF), M4/E4 (25BTF/75RF), M5/E5 (25WTF/25 BTF/50RF), M6/E6 (50WTF/50RF), M7/E7 (50BTF/50RF).

**Table 7:** Pasting properties of native blend flours (M1–M7).

Blends (g 100 g^−1^)	Pasting Viscosity (cp)	Through Viscosity (cp)	Breakdown Viscosity (cp)	Final Viscosity (cp)	Setback Viscosity (cp)	Peak Time (min)	Pasting Temperature (°C)
1 (100RF)	3010 ± 72.06 ^e^	1504 ± 109.01 ^d^	1506 ± 105.47 ^c^	4049 ± 92.57 ^e^	2544 ± 49.52 ^d^	5.6 ± 0.07 ^a^	78.3 ± 0.08 ^a^
2 (12.5WTF/12.5BTF/75 RF)	2141 ± 16.17 ^cd^	1247 ± 24.79 ^c^	893 ± 25.50 ^b^	3154 ± 37.64 ^cd^	1907 ± 26.03 ^c^	5.7 ± 0.03 ^abc^	78.4 ± 0.05 ^a^
3 (25WTF/75RF)	2050 ± 16.00 ^c^	1237 ± 44.16 ^c^	813 ± 57.81 ^b^	3088 ± 24.34 ^c^	1851 ± 32.42 ^c^	5.6 ± 0.04 ^ab^	78.8 ± 0.45 ^a^
4 (25BTF/75RF)	2213 ± 40.04 ^d^	1325 ± 82.42 ^c^	888 ± 66.84 ^b^	3237 ± 44.38 ^d^	1912 ± 38.21 ^c^	5.7 ± 0.00 ^bc^	78.3 ± 0.08 ^a^
5 (25WTF/25BTF/50RF)	1558 ± 16.00 ^a^	1079 ± 17.09 ^ab^	479 ± 2.00 ^a^	2622 ± 10.26 ^b^	1543 ± 8.08 ^b^	5.8 ± 0.04 ^cd^	80.0 ± 0.09 ^a^
6 (50WTF/50RF)	1717 ± 14.22 ^b^	1210 ± 3.00 ^bc^	507 ± 11.93 ^a^	3124 ± 23.52 ^cd^	1914 ± 20.52 ^c^	5.9 ± 0.00 ^d^	85.1 ± 4.48 ^b^
7 (50BTF/50RF)	1523 ± 9.29 ^a^	1048 ± 5.51 ^a^	474 ± 7.09 ^a^	2353 ± 27.50 ^a^	1305 ± 27.50 ^a^	5.7 ± 0.00 ^bc^	79.7 ± 0.51 ^a^

Different letters indicate significant differences (*p* < 0.05) between formulas. M1/E1(100RF), M2/E2 (12.5WTF/12.5 BTF/75RF), M3/E3 (25WTF/75RF), M4/E4 (25BTF/75RF), M5/E5 (25WTF/25 BTF/50RF), M6/E6 (50WTF/50RF), M7/E7 (50BTF/50RF).

**Table 8:** Pasting properties of extruded blend flours (E1–E7).

Blends (g 100 g^−1^)	Cold Peak (cp)	Raw Peak (cp)	Hold (cp)	Breakdown Viscosity (cp)	Final Viscosity (cp)	Setback Viscosity (cp)	Peak Time (min)	Cold Peak Area
1 (100RF)	851 ± 170.41 ^d^	896 ± 160.04 ^cd^	61 ± 11.15 ^a^	835 ± 148.94 ^cd^	302 ± 44.64 ^a^	241 ± 33.50 ^b^	2.1 ± 0.00 ^a^	1830.3 ± 436.56 ^c^
2 (12.5WTF/12.5 BTF/75RF)	641 ± 43.02 ^abc^	642 ± 43.59 ^abc^	75 ± 40.71 ^ab^	567 ± 84.18 ^abc^	263 ± 42.16 ^a^	188 ± 2.08 ^a^	2.2 ± 0.15 ^ab^	1063.2 ± 75.15 ^ab^
3 (25WTF/75RF)	982 ± 67.73 ^cd^	987 ± 64.13 ^d^	111 ± 64.13 ^b^	876 ± 54.53 ^d^	298 ± 10.82 ^a^	187 ± 6.24 ^a^	2.2 ± 0.10 ^ab^	1543.2 ± 83.87 ^bc^
4 (25BTF/75RF)	554 ± 104.20 ^ab^	504 ± 184.92 ^a^	77 ± 5.13 ^ab^	428 ± 180.94 ^ab^	244 ± 28.73 ^a^	167 ± 24.27 ^a^	2.2 ± 0.17 ^ab^	849.3 ± 200.67 ^a^
5 (25WTF/25 BTF/50RF)	693 ± 59.03 ^abcd^	726 ± 56.05 ^abcd^	109 ± 9.50 ^ab^	617 ± 61.88 ^bcd^	317 ± 13.08 ^a^	208 ± 6.03 ^ab^	2.5 ± 0.19 ^bc^	1001.8 ± 73.83 ^ab^
6 (50WTF/50RF)	372 ± 4.93 ^a^	537 ± 7.55 ^ab^	213 ± 8.50 ^c^	324 ± 2.08 ^a^	517 ± 6.51 ^b^	304 ± 3.00 ^c^	6.2 ± 0.04 ^d^	642.2 ± 10.34 ^a^
7 (50BTF/50RF)	762 ± 35.17 ^bcd^	810 ± 28.69 ^bcd^	114 ± 11.50 ^b^	696 ± 32.08 ^bcd^	306 ± 16.50 ^a^	193 ± 5.03 ^a^	2.8 ± 0.14 ^c^	1115.2 ± 124.89 ^ab^

Different letters indicate significant differences (*p* < 0.05) between formulas. M1/E1(100RF), M2/E2 (12.5WTF/12.5 BTF/75RF), M3/E3 (25WTF/75RF), M4/E4 (25BTF/75RF), M5/E5 (25WTF/25 BTF/50RF), M6/E6 (50WTF/50RF), M7/E7 (50BTF/50RF).

## Data Availability

Not applicable.

## Supplementary Materials

The following supporting information can be downloaded at: https://www.mdpi.com/article/10.3390/foods11223610/s1. Figure S1. Viscosimetric and calorimetric parameters evaluated in native (A) and extruded (B) blend flours. Pasting temperature (PT), pasting viscosity (PV), peak time (Pt), breakdown viscosity (BD), hold (H), setback viscosity (SB), final viscosity (FV). Figure S2. Viscosimetric and calorimetric profiles of native blend flours (M1–M7). M1 (100RF), M2 (12.5WTF/12.5BTF/75RF), M3 (25WTF/75RF), M4 (25BTF/75RF), M5 (25WTF/25 BTF/50RF), M6 (50WTF/50RF), M7 (50BTF/50RF). Figure S3. Viscosimetric and calorimetric profiles of extruded blend flours (E1–E7). E1(100RF), E2(12.5WTF/12.5BTF/75RF), E3(25WTF/75RF), E4(25BTF/75RF), E5(25WTF/25 BTF/50RF), E6 (50WTF/50RF), E7(50BTF/50RF).

## References

[1] Yu J.M., Lee J.H., Park J.D., Choi Y.S., Sung J.M., Jang H.W. (2021). Analyzing Gluten Content in Various Food Products Using Different Types of ELISA Test Kits. Foods.

[2] Howdle P.D. (2006). Gliadin, glutenin or both? The search for the Holy Grail in coeliac disease. Eur. J. Gastroenterol. Hepatol..

[3] Shewry P.R., Tatham A.S. (1990). The prolamin storage proteins of cereal seeds: Structure and evolution. Biochem. J..

[4] Thompson T. (1997). Do oats belong in a gluten-free diet?. J. Am. Dietetic Assoc..

[5] Janatuinen E.K., Pikkanainen P.H., Kemppainen T.A., Kosma V.M., Järvinen R.M., Uusitupa M.I., Julkunen R.J. (1995). A comparison of diets with and without oats in adults with celiac disease. N. Engl. J. Med..

[6] Caio G., Volta U., Sapone A., Leffler D.A., De Giorgio R., Catassi C., Fasano A. (2019). Celiac disease: A comprehensive current review. BMC Med..

[7] Prada M., Godinho C., Rodrigues D., Lopes C., Garrido M. (2019). The Impact of Gluten-Free Claim on the Perceived Healthfulness, Calories, Level of Processing and Expected Taste of Food Products. Food Qual. Prefer..

[8] Stoven S., Murray J.A., Marietta E. (2012). Celiac disease: Advances in treatment via gluten modification. Clin. Gastroenterol. Hepatol..

[9] Saturni L., Ferretti G., Bacchetti T. (2010). The gluten-free diet: Safety and nutritional quality. Nutrients.

[10] Niland B., Cash B.D. (2018). Health Benefits and Adverse Effects of a Gluten-Free Diet in Non-Celiac Disease Patients. Gastroenterol. Hepatol..

[11] Cappelli A., Oliva N., Cini E. (2020). A systematic review of gluten-free dough and bread: Dough rheology, bread characteristics, and improvement strategies. Appl. Sci..

[12] Culetu A., Susman I.E., Duta D.E., Belc N. (2021). Nutritional and Functional Properties of Gluten-Free Flours. Appl. Sci..

[13] Gabrovská D., Hálová I., Chrpová D., Ouhrabková J., Sluková M., Vavreinová S., Faměra O., Kohout P., Pánek J., Skřivan P. (2015). Cereals in Human Nutrition, (Obiloviny v Lidské Výživě).

[14] Bultosa G., Taylor J.R.N. (2003). Chemical and physical characterisation of grain tef (Eragrostis tef (Zucc.) Trotter) starch granule composition. Starch/Stärke.

[15] Bultosa G., Wrigley C., Corke H., Seetharaman K., Faubion J. (2016). Teff: Overview. Encyclopedia of Food Grains.

[16] Ketema S. (1997). Tef. Eragrostis tef (Zucc.) Trotter. Promoting the Conservation and Use of Underutilized and Neglected Crops. 12.

[17] Rico D., Ronda F., Villanueva M., Perez Montero C., Martin-Diana A.B. (2019). Development of healthy gluten-free crackers from white and brown tef (*Eragrostis tef* Zucc.) flours. Heliyon.

[18] Mengesha M.H., Pickett R.C., Davis R.L. (1965). Genetic Variability and Interrelationship of Characters in Teff, *Eragrostis tef* (Zucc.) Trotter. Crop Sci..

[19] Ronda F., Abebe W., Pérez-Quire S., Collar C. (2015). Suitability of tef varieties in mixed wheat flour bread matrices: A physico-chemical and nutritional approach. J. Cereal Sci..

[20] Abebe W., Collar C., Ronda F. (2015). Impact of variety type and particle size distribution on starch enzymatic hydrolysis and functional properties of tef flours. Carbohydr. Polym..

[21] Zhu F. (2018). Chemical composition and food uses of teff (*Eragrostis* tef). Food Chem..

[22] Wolter A., Hager A.S., Zannini E., Arendt E.K. (2013). In vitro starch digestibility and predicted glycaemic indexes of buckwheat, oat, quinoa, sorghum, teff and commercial gluten-free bread. J. Cereal Sci..

[23] Gómez M., Martinez M. (2016). Changing flour functionality through physical treatments for the production of gluten-free baking goods. J. Cereal Sci..

[24] Stojceska V., Ainsworth P., Plunkett A., İbanoğlu S. (2010). The advantage of using extrusion processing for increasing dietary fibre level in gluten-free products. Food Chem..

[25] Jafari M., Koocheki A., Milani E. (2017). Functional effects of xanthan gum on quality attributes and microstructure of extruded sorghum-wheat composite dough and bread. LWT—Food Sci. Technol..

[26] Jing Y., Chi Y. (2013). Effects of twin-screw extrusion on soluble dietary fibre and physicochemical properties of soybean residue. Food Chem..

[27] Cheftel J. (1986). Nutritional effects of extrusion-cooking. Food Chem..

[28] Dalbhagat C.G., Mahato D.K., Mishra H.N. (2019). Effect of extrusion processing on physicochemical, functional and nutritional characteristics of rice and rice-based products: A review. Trends Food Sci. Technol..

[29] Kristiawan M., Micard V., Maladira P., Alchamieh C., Maigret J.E., Réguerre A.L., Emin M.A., Della Valle G. (2018). Multi-scale structural changes of starch and proteins during pea flour extrusion. Food Res. Int..

[30] Nikmaram N., Leong S., Koubaa M., Zhu Z., Barba F., Greiner R., Oey I., Roohinejad S. (2017). Effect of extrusion on the anti-nutritional factors of food products: An overview. Food Control.

[31] Robin F., Dubois C., Pineau N., Labat E., Théoduloz C., Curti D. (2012). Process, structure and texture of extruded whole wheat. J. Cereal Sci..

[32] Robin F., Théoduloz C., Srichuwong S. (2015). Properties of extruded whole grain cereals and pseudocereals flours. Int. J. Food Sci. Technol..

[33] Diaz J.M.R., Kirjoranta S., Tenitz S., Penttilä P.A., Serimaa R., Lampi A.M., Jouppila K. (2013). Use of Amaranth, Quinoa and Kañiwa in extruded corn-based snacks. J. Cereal Sci..

[34] Sundarrajan L. (2014). Effect of Extrusion Cooking on the Nutritional Properties of Amaranth, Quinoa, Kañiwa and Lupine. Master’s Thesis.

[35] Nadeesha Dilrukshi H.N., Torrico D.D., Brennan M.A., Brennan C.S. (2022). Effects of extrusion processing on the bioactive constituents, in vitro digestibility, amino acid composition, and antioxidant potential of novel gluten-free extruded snacks fortified with cowpea and whey protein concentrate. Food Chem..

[36] AOAC (2005). Methods 990.03, 2003.05, 985.29 & 923.03. Official Methods of Analysis of AOAC International.

[37] Nelson A.L. (2001). Properties of high-fibre ingredients. Cereal. Foods World.

[38] Beuchat L.R. (1977). Functional and electrophoretic characteristics of succinylated peanut flour protein. J. Agric. Food Chem..

[39] Kaushal P., Kumar V., Sharma H.K. (2012). Comparative study of physicochemical, functional, antinutritional and pasting properties of taro (*Colocasia esculenta*), rice (*Oryza sativa*) flour, pigeon pea (*Cajanus cajan*) flour and their blends. LWT—Food Sci. Tech..

[40] Slinkard K., Singleton V.L. (1977). Total phenol analyses: Automation and comparison with manual methods. Am. J. Enol. Viticult..

[41] Brand-Williams W., Cuvelier M.E., Berset C. (1995). Use of a free radical method to evaluate antioxidant activity. LWT—Food Sci. Tech..

[42] Serpen A., Capuano E., Fogliano V., Gökmen V. (2007). A new procedure to measure the antioxidant activity of insoluble food components. J. Agric. Food Chem..

[43] Ou B., Hampsch-Woodill M., Prior R.L. (2001). Development and Validation of an Improved Oxygen Radical Absorbance Capacity Assay Using Fluorescein as the Fluorescent Probe. J. Agric. Food Chem..

[44] Miller N.J., Rice-Evans C.A. (1997). Factors influencing the antioxidant activity determined by the ABTS^•+^ radical cation assay. Free Radic. Res..

[45] Martin-Diana A.B., Izquierdo N., Albertos I., Sanchez M.S., Herrero A., Sanz M.A., Rico D. (2017). Valorization of Carob’s germ and seed peel as natural antioxidant ingredients in gluten-free crackers. J. Food Process. Preserv..

[46] Pereira J.A., Oliveira I., Sousa A., Ferreira C.F.R., Bento A., Estevinho L. (2008). Bioactive properties and chemical composition of six walnut (*Juglans regia* L.) cultivars. Food Chem. Toxicol..

[47] Gularte M., Rosell C. (2011). Physicochemical properties and enzymatic hydrolysis of different starches in the presence of hydrocolloids. Carbohydr. Polym..

[48] Granfeldt Y. (1994). Foods Factors Affecting Metabolic Responses to Cereal Products. Ph.D. Thesis.

[49] Clerici M.T.P.S., Airoldi C., El-Dash A.A. (2009). Production of acidic extruded rice flour and its influence on the qualities of gluten-free bread. LWT—Food Sci. Technol..

[50] Sivaramakrishnan H.P., Senge B., Chattopadhyay P.K. (2004). Rheological properties of rice dough for making rice bread. J. Food Eng..

[51] Hager A.S., Bormans G.M., Delcour A. (2014). Physical and Molecular Changes during the Storage of Gluten-Free Rice and Oat Bread. J. Agric. Food Chem..

[52] Oppong D., Panpipat W., Chaijan M. (2021). Chemical, physical, and functional properties of Thai indigenous brown rice flours. PLoS ONE.

[53] Ethiopian Health and Nutrition Research Institute (EHNRI) (1998). Food Composition Table for Use in Ethiopia.

[54] Camire M.E., Camire A.L., Krumhar K. (1990). Chemical and nutritional changes. Crit. Rev. Food Sci. Nutr..

[55] Singh S., Gamlath S., Wakeling L. (2007). Nutritional aspects of food extrusion: A review. Int. J. Food Sci. Technol..

[56] Rico D., Cano A.B., Martín-Diana A.B. (2021). Pulse-Cereal Blend Extrusion for Improving the Antioxidant Properties of a Gluten-Free Flour. Molecules.

[57] Ramírez J., Wanderlei C., Meléndez A., Lima O., Penteado M. (2013). Caracterización Físico-Química de Pellets Extruídos de Torta de Higuerilla (Ricinus comunis L.) Visando su uso en Alimentos Balanceados.

[58] Youssef M.K.E., Nassar A.G., EL–Fishawy F.A., Mostafa M.A. (2016). Assessment of proximate chemical composition and nutritional status of wheat biscuits fortified with oat powder. Assiut. J. Agric. Sci..

[59] Frame N.D. (1994). Operational Characteristics of the Co Rotating Twin Screw Extruder. Technology of Extrusion Cooking.

[60] De Pilli T., Giuliani R., Carbone B.F., Derossi A., Severini C. (2005). Study on different emulsifiers to retain fatty fraction during extrusion of fatty flours. Cereal. Chem..

[61] Zhong L., Fang Z., Wahlqvist M.L., Hodgson J.M., Johnson S.K. (2021). Multi-response surface optimisation of extrusion cooking to increase soluble dietary fibre and polyphenols in lupin seed coat. LWT.

[62] Ye G., Wu Y., Wang L., Tan B., Shen W., Li X., Liu Y., Tian X., Zhang D. (2021). Comparison of six modification methods on the chemical composition, functional properties and antioxidant capacity of wheat bran. LWT.

[63] Pérez-Navarrete C., González R., Chel-Guerrero L., Betancur-Ancona D. (2006). Effect of extrusion on nutritional quality of maize and Lima bean flour blends. J. Sci. Food Agric..

[64] Wu N.-N., Ma Z.-Q., Li H.-H., Tian X.-H., Fang Y., Tan B. (2021). Nutritional and cooking quality improvement of brown rice noodles prepared with extruded rice bran. Cereal Chem..

[65] Bhatnagar S., Hanna M.A. (1994). Amylose–lipid complex formation during single-screw extrusion of various corn starches. Cereal Chem..

[66] Nascimento K.D.O.D., Paes S.D.N.D., Oliveira I.R.D., Reis I.P., Augusta I.M. (2018). Teff: Suitability for Different Food Applications and as a Raw Material of Gluten-free, a Literature Review. J. Food Nutr. Res..

[67] Bhatnagar S., Hanna M.A. (1985). Extrusion processing conditions for amylose-lipid complexing. Cereal Chem..

[68] Bekabil F., Befekadu B., Simons R., Tareke B. (2011). Strengthening the Teff Value Chain in Ethiopia. Table off Arm Level Profitability Analysis.

[69] Kuchin N., Sizova Y., Kuleshova L. (2021). The effect of extrusion on the nutrient content of barley as a feed material. IOP Conf. Ser. Earth Environ. Sci..

[70] Ismagilov R., Ayupov D., Nurlygayanov R., Ahiyarova L., Abdulloev V. (2020). Ways to reduce anti-nutritional substances in winter rye grain. Physiol. Mol. Biol. Plants.

[71] Leonel M., Freitas T., Mischan M. (2009). Physical characteristics of extruded cassava starch. Sci. Agric..

[72] Bhattacharya S., Sivakumar V., Chakraborty D. (1997). Changes in CIE Lab colour parameters due to extrusion of rice-green gram blend: A response surface approach. J. Food Eng..

[73] Rosentrater K.A., Muthukumarappan K., Kannadhason S. (2009). Effects of Ingredients and Extrusion Parameters on Aquafeeds Containing DDGS and Potato Starch. J. Aqua. Feed Sci. Nutr..

[74] Paes M.C.D., Maga J. (2004). Effect of extrusion on essential amino acids profile and color of whole-grain flours of Quality Protein Maize (QPM) and Normal Maize Cultivars. Rev. Bras. De Milho E Sorgo.

[75] Jozinović A., Šubarić D., Ačkar D., Babić J., Miličević B. (2016). Influence of spelt flour addition on properties of extruded products based on corn grits. J. Food Eng..

[76] Wang Q., Li L., Zheng X. (2020). A review of milling damaged starch: Generation, measurement, functionality and its effect on starch-based food systems. Food Chem..

[77] Salamoni F., da Costa E., Soares M., Souza J.A., Vânia A. (2014). Physical and functional evaluation of extruded flours obtained from different rice genotypes. Ciência E Agrotecnologia.

[78] Riaz M.N. (2000). Introduction to Extruders and Their Principles. Extruders in Food Applications.

[79] Ding Q., Ainsworth P., Tucker G., Marson H. (2005). The effect of extrusion conditions on the physicochemical properties and sensory characteristics of rice-based expanded snack. J. Food Eng..

[80] de la Hera E., Gomez M., Rosell C.M. (2013). Particle size distribution of rice flour affecting the starch enzymatic hydrolysis and hydration properties. Carbohydr Polym..

[81] Meuser F., Wiedmann W., Mercier C., Linko P., Harper J.M. (1989). Extrusion plant design. Extrusion Cooking.

[82] Veronica A.O., Olusola O.O., Adebowale E.A. (2006). Qualities of extruded puffed snacks from maize/soybean Food Bioprocess Technol mixture. J. Food Proc. Eng..

[83] Onwulata C.I., Konstance R.P., Smith P.W., Holsinger V.H. (2001). Co-extrusion of Dietary Fiber and Milk Proteins in Expanded Corn Products. LWT—Food Sci. Technol..

[84] Bultosa G., Hall A.N., Taylor J.R.N. (2002). Physico-chemical Characterization of Grain Tef [*Eragrostis tef (Zucc.)* Trotter] Starch. Starch-Stärke.

[85] Corke H., Wrigley C., Corke H., Seetharaman K., Faubion J. (2016). Grain: Morphology of Internal Structure. Encyclopedia of Food Grains.

[86] Kumar L., Brennan M., Brennan C., Zheng H. (2022). Influence of whey protein isolate on pasting, thermal, and structural characteristics of oat starch. J. Dairy Sci..

[87] Sayar S., Koksel H., Turhan M. (2005). The Effects of Protein-Rich Fraction and Defatting on Pasting Behavior of Chickpea Starch. Starch-Stärke.

[88] Yang L., Zhang H., Cheng L., Gu Z., Hua D., Qi X., Qian H., Wang L. (2014). Effect of Extrusion on the Hydrophilic Antioxidant Capacity of Four Whole Grains. J. Food Nutr. Res..

[89] Forsido S.F., Rupasinghe H.P.V., Astatkie T. (2013). Antioxidant capacity, total phenolics and nutritional content in selected ethiopian staple food ingredients. Int. J. Food Sci. Nutr..

[90] Shumoy H., Raes K. (2017). Tef: The Rising Ancient Cereal: What do we know about its Nutritional and Health Benefits?. Plant Foods Hum. Nutr..

[91] Hu Z., Tang X., Zhang M., Hu X., Yu C., Zhu Z., Shao Y. (2018). Effects of different extrusion temperatures on extrusion behavior, phenolic acids, antioxidant activity, anthocyanins and phytosterols of black rice. RSC Adv..

[92] Rochín-Medina J.J., Gutiérrez-Dorado R., Mora-Rochín S., Medina-Godoy S., Valdez-Ortiz Á., López-Valenzuela J., Delgado-Vargas F., Milán-Carrillo J., Reyes-Moreno C. (2012). Nutraceutical beverage from a high antioxidant activity mixture of extruded whole maize and chickpea flours. Eur. Int. J. Sci. Technol..

[93] Lindenmeier M., Faist V., Hofmann T. (2002). Structural and functional characterization of pronyl-lysine, a novel protein modification in bread crust melanoidins showing in vitro antioxidative and phase I/II enzyme modulating activity. J. Agric. Food Chem..

[94] Martysiak-Żurowska D., Wenta W. (2012). A comparison of ABTS and DPPH methods for assessing the total antioxidant capacity of human milk. Acta Sci. Pol. Technol. Aliment..

[95] Liu K. (2011). Comparison of Lipid Content and Fatty Acid Composition and Their Distribution within Seeds of 5 Small Grain Species. J. Food Sci..

[96] Ryan E., Galvin K., O’Connor T.P., Maguire A.R. (2007). Phytosterol, Squalene, Tocopherol Content and Fatty Acid Profile of Selected Seeds, Grains, and Legumes. Plant Foods Hum. Nutr..

[97] Martens B.M.J., Gerrits W.J.J., Bruininx E.M.A.M., Schols H.A. (2018). Amylopectin structure and crystallinity explains variation in digestion kinetics of starches across botanic sources in an in vitro pig model. J. Anim. Sci. Biotechnol..

[98] Callejo M.J., Benavente E., Ezpeleta J.I., Laguna M.J., Carrillo J.M., Rodríguez-Quijano M. (2016). Influence of teff variety and wheat flour strength on breadmaking properties of healthier teff-based breads. J. Cereal Sci..

[99] Oh S.H., Kalyani R.R., Dobs A.S., Allen L., Prentice A. (2013). Diabetes Mellitus: Dietary Management. Encyclopedia of Human Nutrition.

[100] Onwulata C.I., Thomas A.E., Cooke P.H., Phillips J.H., Carvalho C.W.P., Ascheri J.L.R., Tomasula P.M. (2010). Glycemic potential of extruded barley, cassava, corn, and quinoa enriched with whey proteins and cashew pulp. Int. J. Food Prop..

[101] Cabrera-Chavez F., Barca A.M.C., Islas-Rubio A.R., Marti A., Marengo M., Pagani M.A., Iamett S. (2012). Molecular rearrangements in extrusion processes for the production of amaranth-enriched, gluten-free rice pasta. LWT—Food Sci. Technol..

[102] Liu H., Guo X., Li W., Wang X., Manman I., Peng Q., Wang M. (2015). Changes in physicochemical properties and in vitro digestibility of common buckwheat starch by heat-moisture treatment and annealing. Carbohydr. Polym..

