# Supplementary material for: Twin-Screw Extrusion as Hydrothermal Technology for the Development of Gluten-Free Teff Flours: Effect on Antioxidant, Glycaemic Index and Techno-Functional Properties
Source: Foods. 2022 Nov 12;11(22):3610. doi: 10.3390/foods11223610 (PMC9689756; doi:10.3390/foods11223610)
Supplement: Supplementary file 1 [file foods-11-03610-s001.zip › foods-2000934-supplementary.pdf]

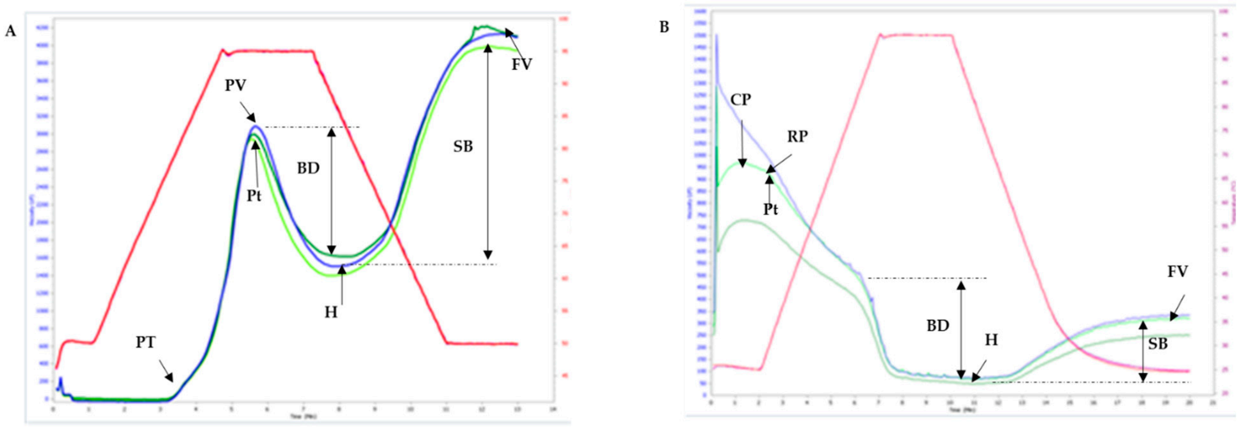

Figure S1. Viscosimetric and calorimetric parameters evaluated in native (A) and extruded (B) blend flours. Pasting temperature (PT), pasting viscosity (PV), peak time (Pt), breakdown viscosity (BD), hold (H), setback viscosity (SB), final viscosity (FV).

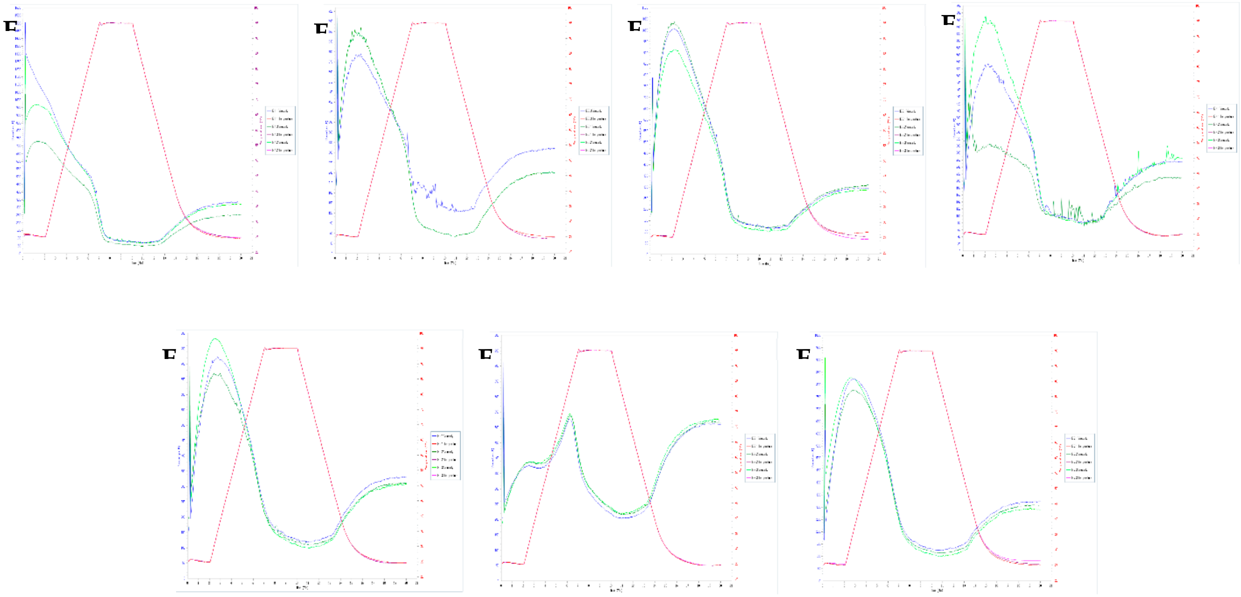

Figure S2. Viscosimetric and calorimetric profiles of native blend flours (M1-M7). M1 (100RF), M2 (12.5WTF/12.5BTF/75RF), M3 (25WTF/75RF), M4 (25BTF/75RF), M5 (25WTF/25 BTF/50RF),M6 (50WTF/50RF), M7 (50BTF/50RF).

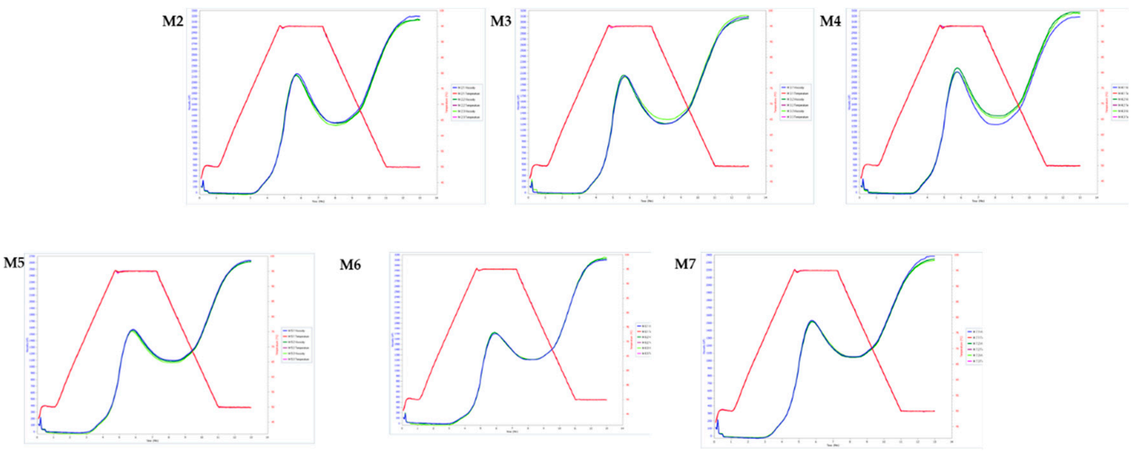

Figure S3. Viscosimetric and calorimetric profiles of extruded blend flours (E1-E7). E1(100RF), E2(12.5WTF/12.5BTF/75RF), E3(25WTF/75RF), E4(25BTF/75RF), E5(25WTF/25 BTF/50RF), E6 (50WTF/50RF), E7(50BTF/50RF).
